# The implicit and explicit attitudes of Chinese university students towards genetic modification

**DOI:** 10.3389/fpsyg.2022.1014395

**Published:** 2022-11-23

**Authors:** Wanyu Zhang, Jilin Zou, Tong Yue

**Affiliations:** ^1^Faculty of Psychology, Southwest University, Chongqing, China; ^2^Department of Psychology, School of Education, Linyi University, Linyi, Shandong, China

**Keywords:** genetic modification, explicit attitude, implicit attitude, single category implicit association test, go/no-go association test

## Abstract

Genetic modification (GM) technology is a technology that changes the characteristics of species through changing the genes of species. Public attitudes toward GM technology have an important impact on the technology’s development. Previous surveys conducted in China used to assess public attitudes toward GM have mostly focused on the explicit level, which is recognized and acknowledged through the self-report method. However, the corresponding research on the implicit level is still lacking, which is unconscious and automated. The public attitudes toward the complete concept of GM are still unclear. In order to fill this gap, this study uses a questionnaire survey (Study 1), and interaction verification of the SC-IAT paradigm and the GNAT paradigm (Study 2) to investigate the explicit and implicit attitudes of Chinese university students towards GM. The role of education level is also examined in this study. The results show that the explicit attitudes of Chinese university students towards GM are generally positive, and the main effect of education level is significant. Finally, the mediating effect of the cognitive level between education level and explicit GM attitude is significant. However, the implicit GM attitudes of university students are generally negative, and neither the main effect of education level nor the mediating effect of cognition level is significant. University students as the future consumers and an important part of public opinion, their attitude to GM will affect the development of GM technology to a large extent. This study provides a theoretical basis for improving Chinese university students’ attitudes toward GM, and also provides new research ideas for the public view of GM.

## Introduction

Genetically modified (GM) technology refers to technology that improves crop varieties, which is achieved by extracting beneficial exogenous genes from donor organisms and introducing them into recipient organisms, or by expressing the silencing endogenous genes in the organism (Kumar et al., 2020). The GM technology contributes to global food security, mitigate climate change-related challenges, and conserve biodiversity. There are also significant productivity, economic, health, and social benefits (James, 2006; ISAAA, 2021). As of 2019, GM crops were used in 71 countries or regions, with a total planting area of 190.4 million hectares. A number of reports have concluded that GM foods currently approved and used on the international market have been risk assessed. The reports showed that the GM foods are unlikely to be a risk to human health (European Food Safety Authority, 2022; GAO, 2002; David et al., 2003; WHO, 2005; James, 2006). In the “30 Years of GM Practice” compiled and published by the Chinese Ministry of Agriculture (2012), it is recorded that China began research on GM crops in the 1980s, and some technologies used in China to cultivate certain crop varieties are more advanced than those used in most other countries. In 2008, the No. 1 Central Document first proposed that China should “start genetically modified organism breeding research.” In June 2009, China issued the “Policy on Promoting the Rapid Development of the Biological Industry,” which mandated “accelerating the construction of the biological industry as a pillar industry in the field of high technology and a strategic emerging industry in China.” Also issued was the Safety Certificate for Production Application (SCPA), which also indicated that China had begun to approve the cultivation of GM crops as food. China now attaches great importance to the cultivation of new GM varieties – and their safety – actively formulating policies to stimulate GM development and establishing a legal system to protect the private rights of GM technology (Ding, 2016).

Public attitudes toward GM technology have had a significant impact on the technology’s development from one saide, the consumer attitudes toward GM safety are a key factor in government policies that are designed to support the development of GM crop industrialization. A meta-analysis has shown that public support for GM technology increases when the potential benefits of GM technology are well expressed, and when the trust of the safety of GM technology increases (Sendhil et al., 2022). Similarly, the studies in China have shown that university students who are familiar with GM have a higher belief in the safety of GM technology and are more willing to support the development and promotion of GM crops (Tang, 2015). Form other side, public’s evaluation of the safety of listed GM foods directly affects their purchase decisions (Ding, 2016), and purchase is also closely related to the future development of GM techology. The group of university students in school is an important part of guiding social opinion. They are also a potential future developer and buyer of GM foods. Therefore, improving university students’ attitudes toward GM can help the development of GM technology to a certain extent. However, public remain cautious about GM technology and the social construction of risk and ethical anxiety raised by GM technology are still controversial issues for the global public (Gaskell et al., 2003; Pang, 2017). For example, people in China who oppose GM technology have argued that GM crops pose huge potential safety risks to human health and the ecological environment (Cui and Yu, 2020). Rumors that GM products have food safety problems and can cause sterility are widespread among the population (Peng and Huang, 2015). Some people even believe that GM technology is a form of bioterrorism against China (Cui and Shoemaker, 2018). Given the spread of similar attitudes among the population, the social acceptance of GM technology in China is low and its development faces great challenges.

In line with the rapid development of GM technology, researchers have been conducting numerous studies on public attitudes toward GM foods. Research has shown that consumer attitudes vary widely from country to country. For example, nearly 70% of American consumers have positive attitudes toward GM foods (Alexander and Schleman, 2003), while consumers in Europe and Japan are more reluctant to purchase GM foods (Burton, 2001; McCluskey et al., 2003). Based on published literature and data analysis, Gu and Li (2021) found that Chinese public attitudes toward GM food safety have fluctuated and changed in recent years, with overall attitudes gradually shifting toward the positive. However, previous researchers have mostly collected information *via* questionnaires and interviews, both of which fall under the category of explicit level measurement. Although self-report methods can better reflect people’s subjective evaluations of their GM attitudes, they may not reflect their true thoughts (Liu and Sang, 2010). This is because humans are susceptible to social approval effects and tend to present themselves in ways they perceive as socially desirable (Fu et al., 2022). In addition, the number of GM questionnaires is huge, and no uniform measurement standard has been established. Other factors, such as researchers’ understanding of GM attitudes and dimension, the test group, and the situation in which the test is conducted will all affect the selection of the questionnaire to a certain degree. The data authenticity of these questionnaires is also limited, to some extent. Wilson et al. (2000) proposed the dual attitudes model, which suggests that people can have two different evaluations of the same attitude object at the same time. One is the explicit attitude that can be recognized and acknowledged by people, and the other is an unconscious, automatically activated implicit attitude. In other words, an individual’s attitude at a certain moment may be implicit, explicit, or the result of both (Zhang and Zhang, 2003). Explicit attitude is the product of individual thinking consciousness and self-reflection and consciousness-level evaluation. Implicit attitude is a product of the unconscious and the unconsciousness-level evaluation. Individuals’ evaluations beyond the conscious level cannot be controlled by themselves and are not influenced by social and cultural factors (Chang and Du, 2007; Yang et al., 2015). According to Epstein’s (1985) Cognitive Experiential Self-Theory (CEST) and Wilson et al.’s (2000) dual attitude model, implicit attitude does not consume mental energy and motivation during retrieval. Thus, they are more reflective of real attitudes and are better able to predict people’s future behavior (Liu and Sang, 2010). Individuals only report episodic attitudes when they retrieve the strength of the episodic attitudes that override and suppress the implicit attitude. When people do not have the ability and motivation to retrieve explicit attitude, they will only report implicit attitude. According to implicit social cognition, even if an individual’s past experiences are not consciously perceived, these experiences may still potentially influence current behaviors and decisions. When an individual’s past GM-related experiences are inconsistent with external sociocultural factors, a separation of external and implicit attitudes occurs (Zhang and Zhang, 2003). Researchers should focus not only on the public’s explicit GM attitudes, also investigate whether GM attitudes have an implicit effect. The first purpose of this study is to explore whether the explicit and implicit attitudes of Chinese university students towards GM are consistent.

A number of studies have also been conducted on the factors that influence public attitudes towards GM, and these studies have been found that there is usually a correlation between education level and GM attitudes. For example, university-educated groups have more positive attitudes and a stronger purchase intention with regard to GM foods (Özel et al., 2010; Jiang et al., 2015; Niu et al., 2019; Wang et al., 2021). Also, most university students are aware of GM technology (Amin et al., 2021). Education, as an important means of socialization, determines the degree of human socialization, and the degree of socialization determines the shaping of behaviors and perceptions (Xu, 2020). Therefore, education’s influence on GM attitudes cannot be ignored. Education is also the main way public acquire GM knowledge. Some studies have shown that the higher the level of cognition about GM, the more receptive individuals to GM technology and GM foods (Huang et al., 2006; Mowen et al., 2007; Fonseca et al., 2012; Tegegne et al., 2013; Xin, 2017; Sheng et al., 2021). For example, courses about GM can be effective in changing students’ attitudes (Troupe et al., 2016); improving public’ cognition of the benefits of agricultural biotechnology can also reduce their negative feelings towards GM (Moon and Balasubramanian, 2001). The results of meta-analysis also reveal the existence of a positive cross-contextual and cross-cultural correlation between GM cognition and attitudes (Allum et al., 2008). Public’ cognition of GM is one of the most important components of their evaluation of GM safety (Zhang and Xu, 2021), and the improvement of publics’ cognition is usually achieved through education. Based on the above studies, an explicit attitude toward GM is not immutable but can be changed to some extent through education. However, no previous research has examined whether implicit GM attitudes are also influenced by education level. The second purpose of this study, therefore, is to explore the effect of education level on implicit attitudes. The results will further reveal the mechanism of explicit attitudes, and provide a reference for improving public attitudes toward GM.

To summarize, this study is designed to: (1) investigate the attitudes of Chinese university students toward GM, and (2) survey the influence of the students’ education level on those attitudes, from both the explicit and implicit aspects. In Study 1, a questionnaire was used to investigate the respondents’ cognitive level of GM and the explicit attitudes of university students with different levels of education. Combined with the previous discussion, the hypothesis put forward after Study 1 is that students with higher education levels have more positive explicit attitudes toward GM. In addition, education level can affect people’s explicit attitudes toward GM by improving cognitive levels. Study 2 explored Chinese university students’ attitudes toward GM at the implicit level. As the traditional Implicit Association Test (IAT) includes two types of attitude objectives, there are multiple possibilities of explanation and uncertainty about the results, and reliable information about the attitudes of theparticipants cannot be obtained (Yao, 2011). Therefore, Study 2 will use the Single Category Implicit Association Test (SC-IAT) and the Go/No-Go Association Test (GNAT) paradigm to verify the implicit effect of GM attitudes. We also investigated whether there are psychological mechanisms consistent with Study 1 at the implicit level of education and cognitive level. This study provides theoretical support for enhancing the attitudes of Chinese university students toward GM technology and also provides new research ideas for public perceptions of GM.

## Study 1: Explicit GM attitude measurements

### Participants

A total of 337 questionnaires were randomly distributed in Southwest University in Chongqing during April to June 2021. After removing the invalid questionnaires due to short response time and duplicate submissions, 318 valid questionnaires were finally collected. There were 211 female students and 107 male students, 200 undergraduate students and 118 graduate students, with an average age of 20.42±3.44 years.

### Measures

The GM Cognition Questionnaire (see Appendix) contains six questions and uses a 5-point scale. The total score shows the participants’ cognitive degree of GM (Guan, 2017). The Cronbach’s *α* = 0.765 > 0.70, and the KMO validity test result was 0.753 > 0.70, which meets the statistical requirements (Zhang and Xu, 2015).

The self-assessment question related to GM explicit attitudes is “What is your level of acceptance of GM technology?” The answers were scored using a 5-point Likert scale. Feng et al. (2012) showed that this single-question questionnaire can also measure reliable data.

### Data analysis

#### Influence of education level on explicit attitude

The statistical results were as follows: 2.20% of the students chose “completely negative,” 7.20% chose “more negative,” 43.40% chose “neutral,” 39.62% chose “more positive,” and 7.55% chose “completely positive.” The mean value was 3.43 ± 0.046, which indicates that the participants’ overall explicit attitude was positive. The results of ANOVA on the explicit attitude index and cognitive level of the participants with different education levels showed that the main effect of education level on the explicit attitude was significant, *F*(1, 317) = 5.473, *p* = 0.020, *η_p_*^2^==0.017. This finding indicates that statistical differences exist in the explicit attitudes of sparticipants with different education levels; The *post hoc* test showed that the difference between the groups of undergraduate and master’s students was significant (*p* = 0.000). The difference between the groups of undergraduate and doctoral students was also significant (*p* = 0.000), while the difference between the groups of master’s and doctoral students was not significant (*p* = 0.064). Therefore, the subsequent study will not distinguish between master’s and doctoral students. The mean value of the explicit attitude of undergraduate students was 3.28 ± 0.783; that of graduate students was 3.69 ± 0.822. This finding indicates that the higher the education level, the more positive the explicit attitude. The main effect of education level was significant, *F*(1, 317) = 7.428, *p* = 0.007, *η_p_*^2^ = 0.023 (undergraduate: 1.10 ± 2.115, graduate: 1.78 ± 2.273). This finding indicates that the higher the education level, the higher the participants’ cognition of GM.

#### Mediating effect analysis

**Figure 1:**
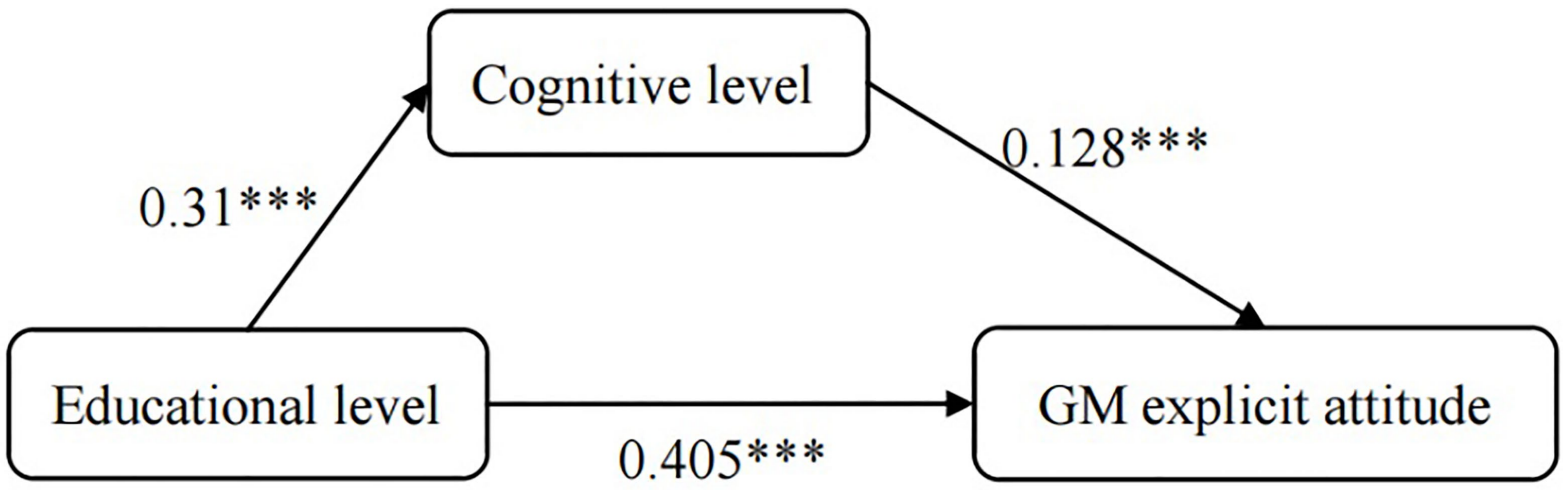
Mediated effect model between cognitive level, education level and explicit attitude. Note: **p < 0.01, ***p < 0.001.

With education level as the independent variable, cognitive level as the mediating variable, and GM cognitive attitude as the dependent variable, a SPSS process script Model 4 was used to conduct a simple mediation effect test. This approach is based on the bootstrap method proposed by Preacher and Halers (Fang and Zhang, 2013) witha 95% confidence interval. The mediating test results show that: (i) the effect of education on the cognitive level, the effect of the cognitive level on GM cognitive attitudes, and the effect of education on GM cognitive attitudes were all significant (*β*_1_ = 0.31, *β*_2_ = 0.128, *β*_3_ = −0.405; *p* < 0.05, see Table 1). This finding indicates that the mediating effect of cognitive level was significant; the mediating effect size was 0.128. (ii) After controlling for the mediating variable (the cognitive level), the cognitive level was significant, and the mediating effect size was 0.128. After controlling for the mediating variable (the cognitive level), the effect of educational level on the level of GM explicit attitude was also significant; the effect size was 0.106 (see Table 2). Therefore, the results indicate that cognitive level plays a mediating role in the effect of educational level on GM explicit attitude level. However, cognitive level was not a complete mediator, but a partial mediator (see Figure 1).

**Table 1:** Mediating effect analysis.

Predictors	Result variables	*β*	*t*	s.e.	*P*	95% confidence interval	
						Upper limit	Lower limit
Educational level	GM attitude	0.405	3.788	0.088	0.000	0.16	0.506
Educational level	Cognitive level	0.31	2.693	0.252	0.007	0.183	1.176
*m*_1_		0.128	6.624	0.019	0.000	0.090	0.166

m_1_: education level – cognitive level – outward attitudes.

**Table 2:** Indirect effects of educational attainment on explicit attitudes through cognitive attainment.

Conditional variables	Effect	Boot SE	Boot LLCL	Boot ULCL
Cognitive level	0.106	0.044	0.025	0.198

### Discussion

The results pertaining to explicit attitudes indicate that Chinese university students’ GM explicit attitudes are generally positive, a finding that is consistent with those of previous studies (Özel et al., 2010; Jiang et al., 2015; Xin, 2017; Niu et al., 2019; Wang et al., 2021). In addition, the main effect of education level is significant, and cognitive level appears to act as a partial mediator between educational level and GM explicit attitude level. This suggests that education level can have a direct effect on GM explicit attitudes; education level can also have an indirect effect on GM explicit attitudes by increasing students’ cognition of GM. Further experiments will measure university students’ GM attitudes at the implicit level, in order to examine whether an implicit effect exists and to verify whether the mediating mechanism of cognitive level can be reproduced in implicit attitudes.

## Study 2: Implicit GM attitude measurements

Using multiple implicit social cognitive tests to study the same psychological phenomenon is a way of complementing different research methods and validating the results obtained at multiple levels. Such methods also make the results more refined, valid and convincing (Zhang and Zhang, 2009; Li et al., 2011). Therefore, the SC-IAT paradigm and the GNAT paradigm will be used to verify the implicit effects of GM attitudes. In addition with regard to the response time index, the SC-IAT paradigm also adds a discriminability index D, which makes up for the limitation of IAT only using response time as an indicator. The SC-IAT paradigm also pays attention to the balance between speed and accuracy (Zhang and Zhang, 2009). The GNAT paradigm focuses on the strength of the linkage between the target category and attributes dimension concepts. This is achieved by comparing the discrimination index D in different tasks, thereby compensating for the limitation of the need to provide category dimensions in IAT experimental designs (Chen, 2015).

“The SC-IAT paradigm and the GNAT paradigm both require four transgenic nouns, positive words and negative words. We used the “nominate first, evaluate later” approach to select the words. (i) in the nomination stage, through a randomly distributed questionnaire, 150 participants were recruited, whose would not participate in the formal experiment of Study 2. They were asked to list five nouns and three positive–negative adjectives related to GM, respectively. The researchers then collected and screened the high-frequency words. (ii) In the evaluation stage, 10 additional informal experiment participants were also randomly selected. They were asked to use a 5-point Likert scale to assess the degree of conformity of each term to GM. The final word list was composed of the top four words with the highest sum of all ratings, as follows: GM food, pest-resistant cotton, GM soybeans, and animal GM (concept words); scientific, productive, high-tech, and innovative (positive attribute words); immature, high-risk, unnatural, and controversial (negative attribute words).

### Experiment A: SC-IAT paradigm

#### Participants

Participants in the current research were randomly recruited from Southwest University. The sample size for the experiment is 70 (12 males and 58 females, including 52 undergraduates and 18 postgraduates; mean age of 20.16 ± 1.85 years old). The software G*Power 3.1 (Faul et al., 2007) was used to set alpha at 0.05, the sample size power at 0.80, and the calculated effect size at 0.69.

#### Procedure

This experiment was a 2 (education level: undergraduate, graduate) × 2 (task type: compatible task, incompatible task) mixed experimental design, with the dependent variables being the participants’ response time and the implicit effect d’ value under different tasks. The E-Prime software program was applied to present the stimulus and record the respondents’ reaction times and accuracy. According to the IAT paradigm, we refer to the task of pairing conceptual words with negative attribute words as a compatibility task, which is usually the opposite of the findings at the explicit level. Thus, in this experiment, the compatibility task was transgenic nouns paired with negative words, and the incompatibility task was GM nouns paired with positive words (Table 3).

**Table 3:** SC-IAT experimental procedures.

Block	Number of stimuli	Function	Reaction	
			Left-click reaction object	Right-click reaction object
1	24	Exercise	GM-related or positive words	Negative words
2	72	Task	GM-related or positive words	Negative words
3	24	Exercise	Positive words	GM-related or negative words
4	72	Task	Positive words	GM-related or negative words

#### Data analysis

According to the method used by Karpinski and Steinman (2006) to process data, this study deleted the data in which the response duration was slower than 350 ms. The response duration of the wrong reaction (false acceptance and false rejection) was replaced with the mean response duration of the correct responses (correct acceptance and correct rejection) of the group to which they belonged, plus 400 ms. Then, the difference between the average response duration of the incompatible task and the compatible task, respectively, was calculated and divided by the standard deviation of all response duration. The obtained value is the implicit effect value d’.

#### Results

**Figure 2:**
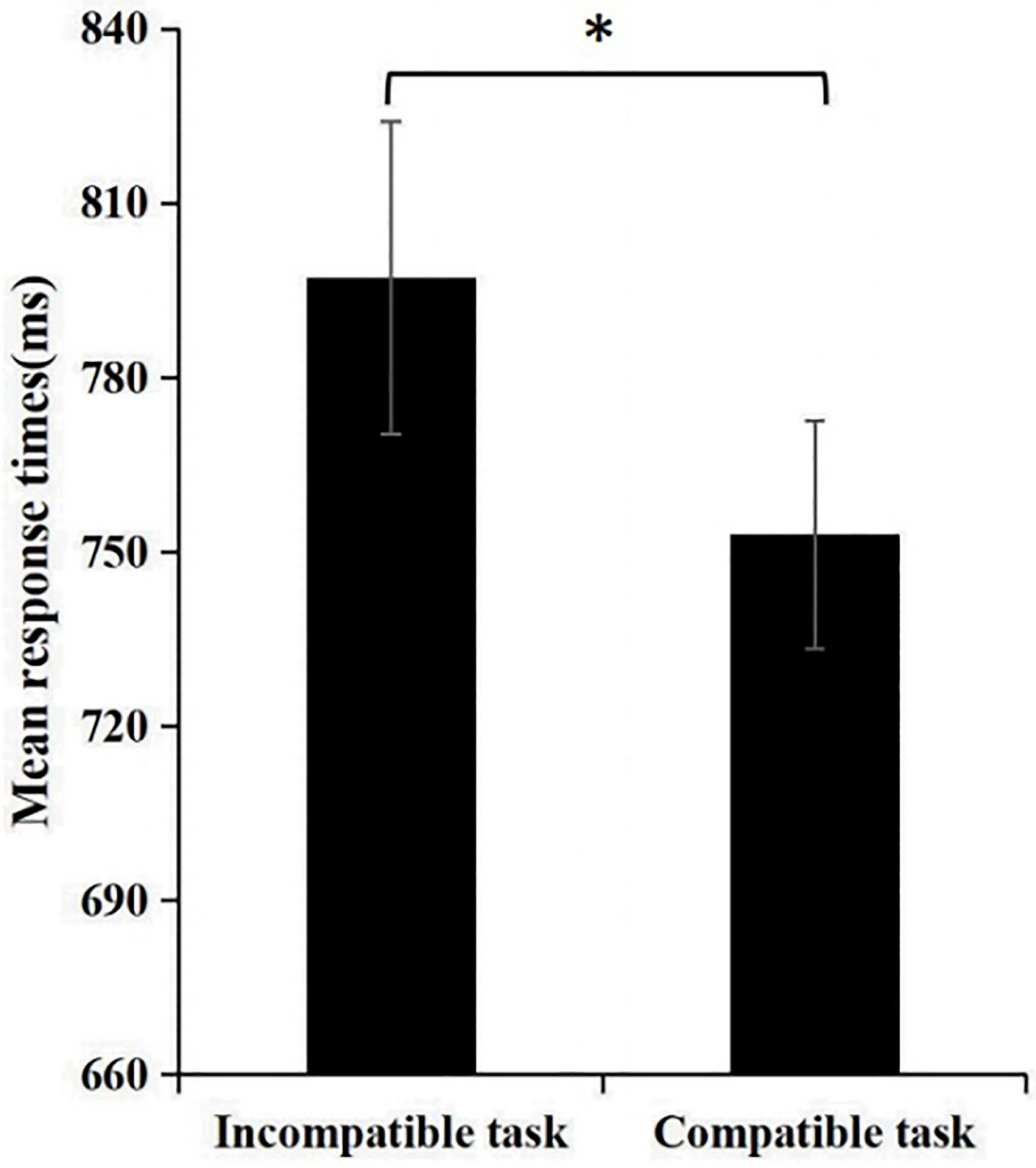
Comparison of compatible and incompatible reactions in SC-IAT. Note: **p < 0.01, ***p < 0.001.

The paired t-test reveals that participants’ response duration on the compatibility task were significantly lower than those on the incompatibility task (*t* = 2.201, *p* = 0.031 < 0.050; see Figure 2). The participants’ mean response duration in the incompatibility task (GM paired with positive words) was 797.11 ± 225.24 ms. In the compatibility phase (GM paired with negative words) the mean response duration were 752.99 ± 164.70 ms. This indicates that participants were more inclined to associate GM with negative attribute words. The greater the discriminative power (d’), the more negative the participants’ implicit attitude towards GM. Conversely, the lower the discriminatory power, the more positive the participants’ implicit attitude. A one-sample t-test for implicit effects was conducted with 0. The results show that *t* = −2.201, *p* = 0.031 < 0.050, and the d’ value reached a significant level, with a mean value of −0.223 ± 0.848.

The independent *t*-test samples for the value of the d’ for participants with different education levels show that the main effect of education level was not significant [*F*(1, 69) = 0.251, *p* = 0.618, undergraduate: −0.201 ± 0.12, and graduate: −0.29 ± 0.19]. This finding indicates that no significant difference exists between the implicit effects of participants with different education levels. With education level as the independent variable, the cognitive level as the mediating variable, and d’ as the dependent variable, the results of the mediation effect test show that the effects of education level on the cognitive level, the effect of the cognitive level on GM implicit attitudes, and the effect of the education level on GM implicit attitudes were not significant. These findings indicate that the mediation effect of the cognitive level was not significant (see Table 4).

**Table 4:** Mediating effect analysis (SC-IAT).

Predictors	Result variables	*β*	*t*	s.e.	*P*	95% confidence interval	
						Upper limit	Lower limit
Education level	Implicit attitude	−0.167	−0.601	0.236	0.550	−0.613	0.329
Education level	Cognitive level	0.406	1.497	0.485	0.139	−0.2418	1.6948
*m*_2_		0.16	1.305	0.580	0.196	−0.401	0.1915

m_2_: educational level – cognitive level – implicit attitude (SC-IAT).

#### Discussion

The SC-IAT results show that the implicit GM attitudes are generally negative among university students. This finding indicates that an implicit effect of GM attitudes exists under the SC-IAT paradigm. In addition, the main effect of education level was not significant, and the mediating effect of the cognitive level was not significant. These findings were inconsistent with the results of explicit attitude, suggesting that the pathways of the effects of education level and cognitive level on explicit and implicit attitudes may be different. In this study, the GNAT paradigm will be used for further measurement, in order to verify the results of this experiment.

### Experiment B: GNAT paradigm

#### Participants

Participants in the current research were randomly recruited from Southwest University. The sample size for this experiment is 64 (30 males, 34 females, including 50 undergraduates and 14 graduate students, with a mean age of 19.86 ± 1.55 years old). The G*Power 3.1 software (Faul et al., 2007) was used to set the alpha at 0.05 and the sample size power at 0.80. The effect size was calculated at 0.457.

#### Procedures

The current experiment was a 2 (education level: undergraduate, graduate) × 2 (lexical association type: GM + positive words, GM + negative words) mixed experimental design. The dependent variables were the participants’ discrimination index D′. The experimental materials were the same as in experiment A, with the signal and noise stimuli redistributed. In this experiment, the first stage of the test required participants to respond to the GM nouns and positive words as signal stimuli (GO), while the GM nouns and negative words were used as noise stimuli, to which the participants were not required to respond (No-Go). The second stage of the test required the opposite. The procedure is shown in Table 5.

**Table 5:** GNAT experimental procedures.

Block	Number of stimuli	Function	Reaction	
			Key response object	Non-responsive objects
1	24	Exercise	GM noun + positive word	GM noun + negative word
2	72	Task	GM noun + positive word	GM noun + negative word
3	24	Exercise	GM noun + negative word	GM noun + positive word
4	72	Task	GM noun + negative word	GM noun + positive word

#### Data analysis

Following the methods of Nosek and Banaji (2001) and He and Liang (2010), the discrimination index D′ was used as the dependent variable. The hit rate and false alarm rate were calculated and converted into the Z-score, and the difference of the Z-score was the value of d’. The larger the value of d’ was, the stronger was the association of the words. The difference between the different lexical association types of discrimination index D′ was taken as the implicit discrimination index D′.

#### Results

**Figure 3:**
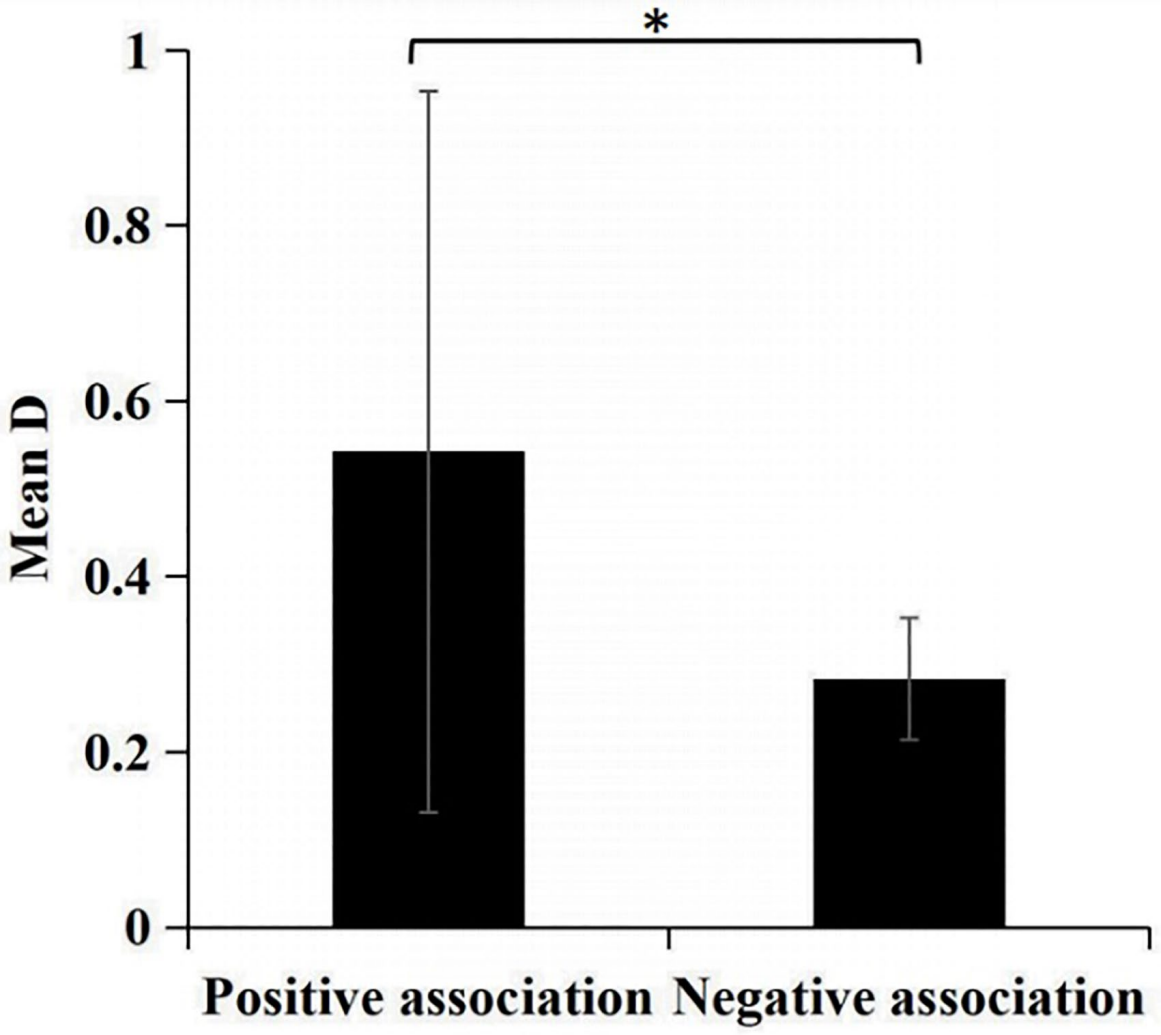
Comparison of the response sensitivity of negative and positive association in GNAT. Note: **p < 0.01, ***p < 0.001.

The paired t-test reveals that the d’ of negative words was significantly higher than that of positive words (*t* = −2.104, *p* = 0.039 < 0.050), with a mean effect value of −0.542 ± 3.290 for negative associations and −0.283 ± 0.552 for positive associations (see Figure 3). This finding means that, when participants used “GM noun + negative attribute word” as a signal, it was easier to distinguish them from the noise, further indicating that participants were more inclined to associate GM with negative attribute words.

An independent sample *t*-test was conducted on the implicit discrimination index D′ of participants with different education levels. The results show that the main effect of education level was not significant [*F*(1, 62) = 6.505, *p* = 0.107, undergraduate: −0.899 ± 0.44, and graduate: −0.223 ± 0.44]. With education level as the independent variable, the cognitive level as the mediating variable, and the D′ as the dependent variable, the results of the mediating effect test showed that the effects of education level on cognitive level, the effect of the cognitive level on implicit attitudes, and the effect of education level on implicit attitudes were not significant. These findings indicate that the mediating effect of cognitive level was not significant (see Table 6).

**Table 6:** Mediating effect analysis (GNAT).

Predictors	Result variables	*β*	*t*	s.e.	*P*	95% confidence interval	
						Upper limit	Lower limit
Education level	Implicit attitude	−0.484	−0.601	0.942	−1.612	−3.404	0.366
Education level	Cognitive level	0.052	0.17	0.705	0.865	−1.290	1.53
*m*_3_		−0.069	1.305	0.170	−0.550	−0.433	0.246

m_3_: educational level – cognitive level - implicit attitude (GNAT).

#### Discussion

The GNAT data show that the implicit discrimination index D was significantly higher when “GM noun + negative attribute” was used as the target stimulus than when “GM noun + positive attribute” was used. This finding indicates that the association between GM nouns and negative attributes was stronger. In addition, this experiment again verified that neither the main effect of education level nor the mediating effect of the cognitive level was significant. The results of the present study and the SC-IAT study validate each other, fully demonstrating that GM attitudes do have the implicit characteristics of automation and unconsciousness.

## General discussion

### University students’ explicit and implicit attitudes towards GM

At the explicit level, the explicit attitudes of university students toward GM were generally positive, which was consistent with most studies (Özel et al., 2010; Wang et al., 2021; Jiang et al., 2015; Xin, 2017; Niu et al., 2019). Meanwhile, at the implicit level, in both paradigms the tendency to pair GM nouns with negative words indicated that the overall implicit attitudes of university students were negative. The results of both studies suggested that the implicit and explicit attitudes of university students were separated, which means that there was an implicit effect of transgenerational attitudes. In the SC-IAT paradigm, participants’ reaction times on compatible tasks were significantly slower than those on incompatible tasks. Participants were more likely to associate GM with negative attribute words. Similarly, in the GNAT paradigm, the association response sensitivity to negative words was significantly higher than to positive words. This finding indicateed that, at the implicit level, the association between GM nouns and negative attribute words was stronger.

In this study, the attitudes of Chinese university students toward GM were investigated from two aspects (explicit and implicit). The results of implicit and explicit levels were found to be inconsistent, which means there was an implicit effect on Chinese university students’ attitudes towards GM. From the analysis of the essential characteristics of implicit and explicit attitudes, the two types of attitudes are detected by two different internal psychological structures, each with different mental processing mechanisms. Individuals’ implicit attitude evaluations of GM do not change as a result of thinking, feeling, or behavior, because implicit attitude is an evaluation at the extra-conscious level. In contrast, individuals’ explicit attitude is an evaluation at the conscious level, which is susceptible to interference from external factors and self-concealment. Students’ outward evaluations of GM are inevitably disturbed and influenced by positive sociocultural factors, such as social opinion, national policy guidance, or school science popularization. Thus, most surveys, including the one used this study, have reported generally positive outward attitudes toward GM. However, at the implicit level, students in higher education did not really recognize the positive effects of GM, and such external socio-cultural factors only acted at the individual level of consciousness.

According to Epstein’s (1985) cognitive-experiential self theory (CEST) and the dual attitude model of Wilson et al. (2000), individuals’ attitudes are related to the cognitive resources they allocate to the subject during the evaluation process. In Study 1, when measuring explicit attitudes, participants had sufficient cognitive resources to engage in self-reflection. This self-aware control shielded the implicit appraisal system, so that the explicit attitudes reflected the influence of externally-imposed factors, such as social culture. In Study 2, participants were asked to respond quickly to stimulus words or word associations, while balancing speed and accuracy. This required more cognitive resources and prevented the participants from self-reflecting and extracting past experiences. When an individual’s past experiences are inconsistent with the external environment, the separation of their external and implicit attitudes occurs.

### Influence of education level on GM attitudes

At the explicit level, the main effect of education was significant. The degree was higher, and the explicit attitude was more positive. In addition, the mediating effect of cognition between education and explicit attitudes was significant. In other words, education has a direct effect on the explicit attitudes of GM, and also has an indirect effect on the explicit attitudes of GM by increasing the cognition of GM among university students. In both implicit paradigms, the main effect of education was not significant; no significant mediating effect of cognitive attainment was found between education and implicit attitudes. This finding indicated that the mechanism of education level is different from that of implicit attitude.

Education, as an important means of socialization, can determine the behavior of individuals and shape their perceptions to a certain extent. Previous studies have also shown that education has a significant effect on GM episodic attitudes (Özel et al., 2010; Wang et al., 2021; Jiang et al., 2015; Niu et al., 2019). However, this effect is limited and cannot really act on individuals’ implicit attitudes. Combined with the dual attitude model, whether acting directly on GM explicit attitudes or indirectly by increasing cognitive levels, education is an externally-imposed sociocultural influence. Notably, education requires significant cognitive resources to be consumed to initiate an impact on individuals. When measuring explicit attitudes, participants have sufficient cognitive resources to mobilize past experiences and self-evaluate. The consciously-controlled explicit attitude system suppresses the implicit system in such a way that, on the one hand, education can have a direct effect on GM explicit attitudes. On the other hand, when measuring implicit attitudes, limited cognitive resources are already occupied, because participants are required to balance speed and accuracy during the implicit paradigm task. It is also difficult to consciously extract the knowledge and concepts acquired through education and to be self-reflective, so influencing GM implicit attitudes by the level of education is difficult.

Most previous studies have shown that more educated groups have more positive explicit attitudes toward GM, which is consistent with the findings of Study 1. From the implicit level, this study also showed that the influence of education level on GM attitudes was limited, as perceptions beyond the consciousness level cannot be shaped by education alone. This process needs to be moderated or combined with other variables. For example, the effect of education on GM attitudes may also be influenced by other factors, such as the government’s supervision and management system, the current state of the industrialization of GM crops, and the credibility of the public sector and the media. A comparison of the GM safety management systems in China and the United States showed that the United States has one of the most comprehensive GM management systems and that American consumers generally trust the regulatory authorities’ decisions on GM foods (Xiao et al., 2008). However, the Chinese government’s GM supervision and management system is relatively backward, with a late start in the construction of relevant laws and regulations, and the lack of a unified management and coordination mechanism. In summary, education alone is not enough to improve the attitudes of university students toward GM. The Chinese government can further improve the credibility of regulatory agencies and learn from the U.S. system of GM biotechnology supervision and management.

## Limitations and prospects

Although this study makes some important findings, there are also some deficiencies. Firstly, the participants in this study were university students, so follow-up studies should increase the number of participants group types to further validate the results. Second, the implicit effect of GM attitudes found in this study can be also explained from other perspectives. In addition to the CEST and dual attitude models, Jones and Davis’s (1965) attribution theory suggests that intrinsic motivation and personality traits are the causes of behavior. The correlation between implicit and explicit attitudes has also been shown to depend on the degree of cognitive thinking. Individuals who tend to be thoughtful also have a lower correlation between implicit and explicit measures (Chen, 2015). According to these theories, some personality traits of university students may also have an impact on the observation of implicit effects. This could introduce new variables for subsequent studies.

Previous studies on GM attitudes have used questionnaire or interview-based measures at the explicit level, and due to the different measurement methods, the results have been inconsistent. In this study, the SC-IAT paradigm and GNAT paradigm were used to verify the implicit effect of GM attitude andexplored the influence of education leve. Both the SC-IAT paradigm and the GNAT paradigm used the implicit discrimination index D′ as the indicator. This approach compensates for the limitations of the traditional IAT paradigm, which only uses response time as an indicator while ignoring information about the error rate.

The speed-accuracy balance is also available.

## Conclusion

This study uses a questionnaire survey (Study 1) and the SC-IAT and GNAT paradigms (Study 2) to examine Chinese university students’ explicit and implicit attitudes toward GM and explored the role of education level. Study 1 shows that Chinese university students have a positive explicit attitude, and the main effect of education level is significant. The partial mediating effect of cognitive level between education level and explicit attitude is also significant. Study 2 shows that the overall implicit attitude of Chinese university students is negative and the main effect of education in this study was not significant. This study was conducted through the combination of implicit and explicit methods showing that there is an implicit effect on the attitudes of Chinese university students toward GM. This study provides a new way to study public perceptions of GM and may stimulate society to think more deeply about GM issues. It also suggests that the influence of education level on GM attitudes is limited, and that perceptions beyond the level of consciousness cannot be changed simply by increasing education level. Rather, this process requires a combination of factors such as government oversight. This provides theoretical support for improving the attitudes of Chinese college students toward GM.

## Data availability statement

The original contributions presented in the study are included in the article/Supplementary material, further inquiries can be directed to the corresponding authors.

## Ethics statement

The studies involving human participants were reviewed and approved by Ethics Committee of Southwest University. The patients/participants provided their written informed consent to participate in this study.

## Author contributions

WZ carried out the experiment and wrote the manuscript with support from TY. JZ and TY helped to support the project. All authors contributed to the article and approved the submitted version.

## Funding

This study was supported by Youth Innovation Team Development Plan in Shandong Province (2019) and the PhD research startup foundation of Southwest University (swu118092).

## Conflict of interest

The authors declare that the research was conducted in the absence of any commercial or financial relationships that could be construed as a potential conflict of interest.

## Publisher’s note

All claims expressed in this article are solely those of the authors and do not necessarily represent those of their affiliated organizations, or those of the publisher, the editors and the reviewers. Any product that may be evaluated in this article, or claim that may be made by its manufacturer, is not guaranteed or endorsed by the publisher.

## Appendix

GM knowledge questionnaire: (a) “GM crops and foods have unpredictable consequences (incorrect).” (b) “There are ecological hazards of GM crops (incorrect).” (c) “GM foods are potentially harmful to human health (incorrect).” (d) “GM crops and foods can help solve the problem of global hunger (correct).” (e) “The development of GM can provide cheaper food for consumers (correct) “. (f) “GM crops and foods can provide people with higher nutrition and value (correct)” (Guan, 2017).
